# Relevance of the Immunohistochemical Expression of p53 and E-cadherin in the Grading of Urothelial Carcinoma: A Single-Center Cross-Sectional Observational Study

**DOI:** 10.7759/cureus.72132

**Published:** 2024-10-22

**Authors:** Mayank Singh, Renu Sahay, Kapil Tiwari, Surya Prakash

**Affiliations:** 1 Department of Pathology, Maharani Laxmi Bai Medical College, Jhansi, IND; 2 Department of Surgery, Maharani Laxmi Bai Medical College, Jhansi, IND

**Keywords:** bladder cancer, e-cadherin, high grade, low grade, p53, urothelial carcinoma

## Abstract

Background

Urothelial carcinoma (UC) is a prevalent cancer worldwide, primarily affecting the urinary bladder. It is more common in men than women and is often linked to factors like tobacco smoking, occupational exposure, and chronic infections. UC can be classified into different subtypes based on its growth pattern (papillary or non-papillary) and the extent of invasion into the bladder wall. The management of UC depends on its grade and stage, with treatment options ranging from transurethral resection of bladder tumor (TURBT) for non-invasive tumors to cystectomy for muscle-invasive disease. This study aimed to investigate the expression of p53 and E-cadherin in low-grade and high-grade UC and their correlation with histomorphological parameters.

Materials and methods

A cross-sectional observational study was conducted on 50 histopathologically confirmed UC cases. Immunohistochemical (IHC) staining for p53 and E-cadherin was performed, and the results were correlated with clinicopathological features. Statistical analysis was performed to find the correlation between p53 and E-cadherin immunoexpression and the grade of UC.

Results

The cohort's mean age was 58.18±12.99 years, with a male predominance (76%). Most cases (52%) were from rural areas and unskilled workers (52%). Hematuria was present in all cases (100%), while urgency and frequency were reported in 32% each. High-grade UC was more common (76%), with 48% being invasive. Muscle invasion was absent in 56%. p53 overexpression was seen in all cases, with 38% moderate and 36% strong staining. Twenty-two out of 24 cases (91.7%) of invasive UC showed low E-cadherin expression, while all 26 cases (100%) of low-grade papillary UC and high-grade papillary UC displayed high E-cadherin expression. This suggests that reduced E-cadherin expression is strongly associated with invasive UC, potentially as a tumor aggressiveness and progression marker.

Conclusion

Considering that elevated p53 protein expression is linked to aggressive tumor behavior, a more intensive treatment strategy is recommended for patients with non-muscle-invasive bladder cancer (NMIBC) who show high p53 protein scores. On the other hand, the downregulation or absence of E-cadherin expression has been recognized as a strong indicator of advanced grade and higher clinical stages.

## Introduction

Urothelial carcinoma (UC), a prevalent and often fatal malignancy, primarily affects the urinary bladder in individuals over 50, with a higher incidence in men [1]. It ranks as the ninth most common cancer globally and seventh among men [2]. In India, the annual occurrence rate is 2.25% per 100,000, with a higher rate in males [3,4].

Most bladder cancers originate from the transitional epithelium (UCs), though squamous and glandular carcinomas also exist [5]. The development of UC is multifactorial, with tobacco smoking, occupational exposures, analgesic abuse, genetic predisposition, and chronic infections playing significant roles [6]. Chemical exposure, particularly in industrial settings associated with petrochemicals and arylamines, is strongly associated with an increased risk [7].

UC can be classified into papillary and non-papillary subtypes, each with distinct genetic underpinnings. Non-invasive papillary carcinomas are more common initially, but a significant proportion can progress to invasive disease [8]. Carcinoma in situ (CIS), a high-grade preinvasive lesion, is often linked to high-grade papillary UC [9].

Treatment of UC depends on its histological grade and stage. Non-invasive papillary tumors are typically managed with transurethral resection of bladder tumor (TURBT), sometimes accompanied by adjuvant intravesical therapy. More invasive tumors may necessitate cystectomy, and the role of neoadjuvant and adjuvant chemotherapy remains under investigation [10].

Accurate diagnosis of UC can be challenging due to its morphological diversity and overlap with other malignancies [11]. Determining the extent of tumor involvement is crucial for treatment planning and prognostication, but microscopic diagnosis alone may not always be sufficient. Therefore, there is a need for additional diagnostic tools to enhance accuracy and improve patient management. This study aims to investigate the potential of E-cadherin and p53 immunohistochemical (IHC) markers as ancillary diagnostic tools in UC.

## Materials and methods

A cross-sectional observational study was conducted at Maharani Laxmi Bai Medical College, Jhansi, on 50 histopathologically confirmed cases of UC between July 2022 and January 2024. The sample size was calculated using a 95% confidence interval and a 4% margin of error. Written informed consent was obtained from all participants, and the study was granted ethical approval by the Institute Ethics Committee (Human Studies) of Maharani Laxmi Bai Medical College, Jhansi (approval number: 2240/IEC/I/2022-23), and all participants gave informed consent before participating in the study. There was no additional cost to our participants. The following inclusion and exclusion criteria were applied for the selection of cases: for inclusion criteria, all histopathologically confirmed cases of UCC and willingness to participate, as indicated by signing informed consent by the patient, and for exclusion criteria, patients with non-malignant lesions (like papilloma, inverted papilloma, or papillary urothelial neoplasm of low malignant potential) or malignancies other than UC (like metastatic carcinoma) and those who declined participation. 

Histopathological analysis

**Figure 1:**
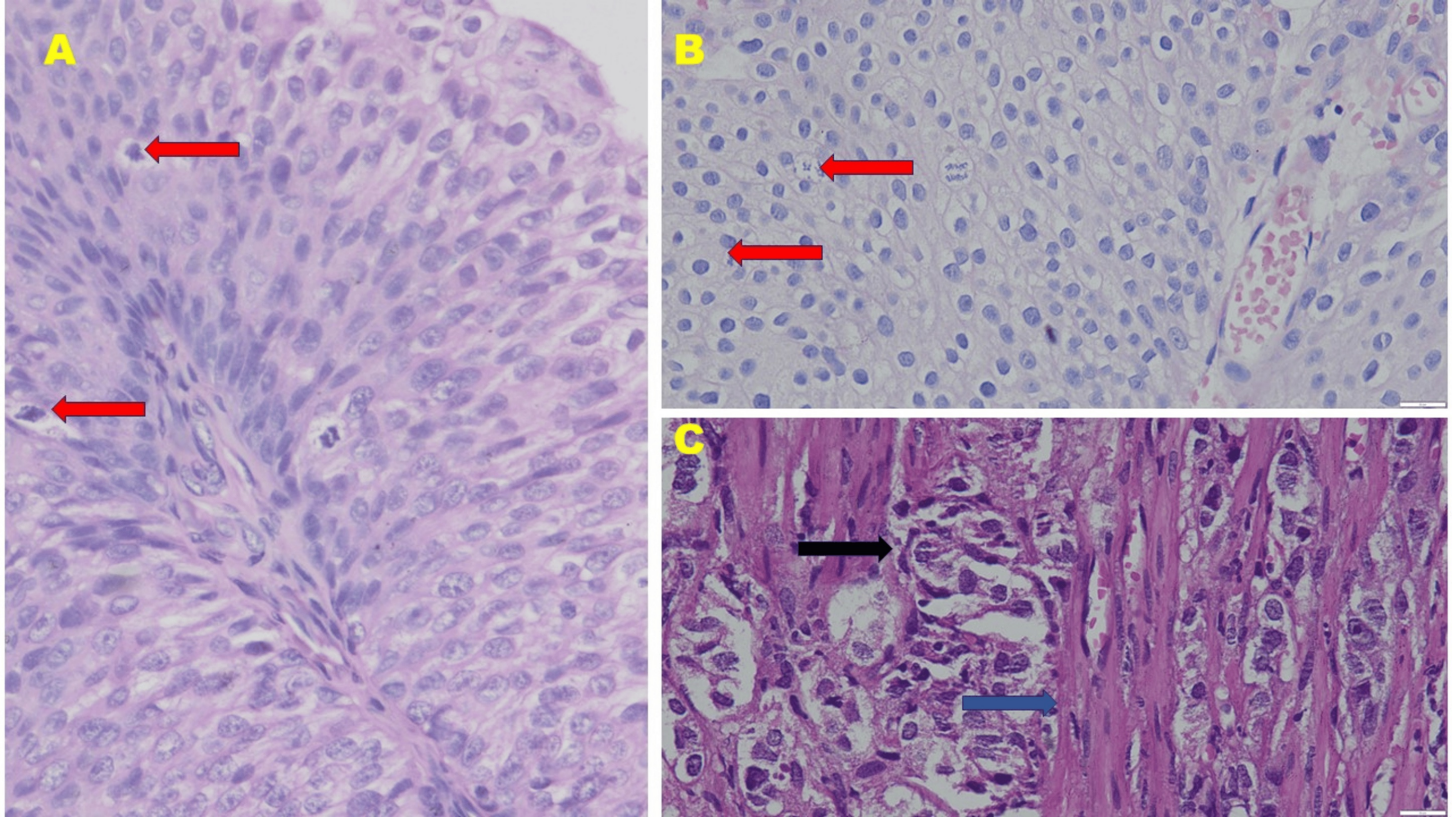
Histomorphology of (A) low-grade papillary urothelial carcinoma, (B) high-grade papillary urothelial carcinoma, and (C) invasive urothelial carcinoma (hematoxylin and eosin stain, 400× magnification) Note the atypical mitotic figures (shown by red arrow) in (A) low-grade papillary urothelial carcinoma and (B) high-grade papillary urothelial carcinoma. Also, note the invasion of malignant urothelial cells (black arrow) between the muscular layer (blue arrow) in (C) invasive urothelial carcinoma

Formalin-fixed paraffin-embedded blocks were retrieved for analysis. Hematoxylin and eosin (H&E) staining was conducted, and the slides were rescored by two expert pathologists to make a histopathological diagnosis of UC based on morphological features. The diagnosis was supported by immunoreactivity assessments for E-cadherin and p53. Histological subclassification of UC was made following WHO criteria, dividing cases into three groups: invasive UC, low-grade papillary UC, and high-grade papillary UC. A representative image of each group is shown in Figure 1. 

IHC analysis

**Figure 2:**
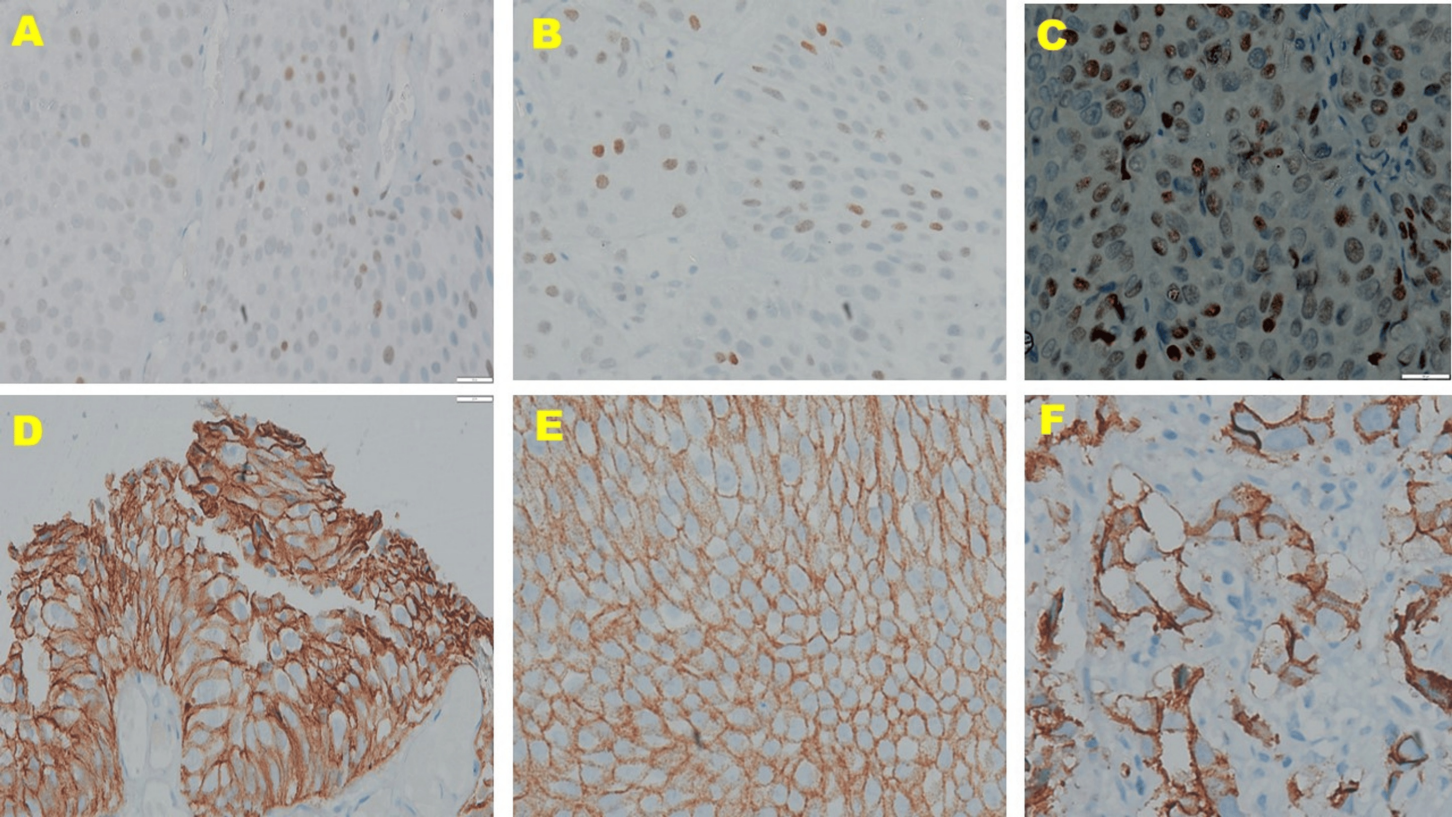
Immunohistochemical expression of p-53 (A, B, C, 100× magnification) and E-cadherin (D, E, F, 100× magnification) in low-grade papillary urothelial carcinoma, high-grade papillary urothelial carcinoma, and invasive urothelial carcinoma, respectively Note the increase in intensity of p-53 immunoreactivity from low-grade papillary urothelial carcinoma (A) to high-grade papillary urothelial carcinoma (B) to invasive urothelial carcinoma (C). In contrast, immunoreactivity to E-cadherin gradually decreases from low-grade papillary urothelial carcinoma (D) to high-grade papillary urothelial carcinoma (E) to invasive urothelial carcinoma (F)

Representative tumor sections were processed for the IHC analysis of E-cadherin and p53 using an in-house automated IHC stainer (Ventana BenchMark GX, Roche Diagnostics, Risch-Rotkreuz, Switzerland). Paraffin-embedded tissue blocks were cooled on a cold plate at 4°C for 20-30 minutes. Then 3-4-μm-thick sections were cut and placed onto poly-L-lysine-coated slides, labeled, and dried at room temperature for 12 hours before further processing. The slides were put on a hot plate at 70°C for 15-20 minutes. Primaries consisted of a ready-to-use anti-E-cadherin (36) mouse monoclonal antibody (Ventana, USA) and a ready-to-use anti-p53 antibody (CONFIRM DO-7, Ventana, USA) (Figure 2).

Statistical analysis

Statistical analysis was performed using IBM SPSS Statistics for Windows, Version 27.0 (Released 2020; IBM Corp., Armonk, New York, United States). Continuous variables are expressed as mean±standard deviation, while categorical variables are presented as frequencies and percentages (n%). The chi-squared test was employed for the comparison of categorical variables, with a significance level set at p<0.05.

## Results

Table 1 shows the detailed analysis of the demographic and clinical profile of 50 cases studied. Interestingly, 14 cases (28%) lie in the age group of 61-70 years followed by 11 cases (22%) in the 51-60-year range, and the least represented age group was 30-40 years comprising only 14% of the total. The mean age of the subjects was 58.18±12.99 years, indicating a predominance of cases among individuals aged over 51 years. Gender distribution revealed that 38 cases (76%) were male, whereas 12 cases (24%) were female, resulting in a male-to-female ratio of 3.17:1. Furthermore, a majority of cases were from rural areas (52%), as opposed to urban settings (48%). The table also presents details of the symptomatic presentation of the cases with 100% hematuria occurrence followed by urgency and frequency, which were reported in 32% of cases, signifying an increased need to urinate. In the histological grading of UC, the authors concluded that 24 cases were invasive UC (48%), 14 cases were high-grade papillary UC (28%), and 12 cases were low-grade papillary UC (24%). 

**Table 1:** General characteristics of the study ^1^Includes workers who possess no particular skill and likely have no formal education like farmers, security guards, construction workers, etc. ^2^Includes workers who require more skills than an unskilled labor job like driver, electrician, machine operator, etc. ^3^Includes workers who have advanced training and formal education in the field concerned like teachers, doctors, nurses, etc.

Parameters		No.	%
Age (in years)	30-40	7	14
	41-50	8	16
	51-60	11	22
	61-70	14	28
	>70	10	20
	Total	50	100
	Mean±SD	58.18±12.99	
Gender distribution	Male	38	76
	Female	12	24
	Total	50	100
Residential status	Rural	26	52
	Urban	24	48
	Total	50	100
Occupation	Unskilled^1^	26	52
	Semi-skilled^2^	17	34
	Professionals^3^	7	14
Symptoms	Hematuria	50	100
	Urgency	16	32
	Frequency	16	32
Histological grade	Invasive urothelial carcinoma	24	48
	Low-grade papillary urothelial carcinoma	12	24
	High-grade papillary urothelial carcinoma	14	28
	Total	50	100

p53 expression

Table 2 shows that 13 cases out of 24 histologically invasive UCs had strong p53 staining, while in 10 cases, moderate p53 staining was observed. In only one case weak p53 staining intensity was observed. In low-grade papillary UC, six cases showed weak and six cases showed moderate p53 staining. Out of 14 cases of high-grade papillary UC, six, three, and five cases showed weak, moderate, and strong p53 staining, respectively. The difference was statistically significant in the statistical analysis.

**Table 2:** Association between histological grade and p53 staining intensity ^1^Chi-squared test *: significant (p<0.05)

Histological grade	p53 staining intensity			P-value^1^
	Weak (n=13)	Moderate (n=19)	Strong (n=18)	
Invasive urothelial carcinoma (n=24)	1	10	13	0.006728*
Low-grade papillary urothelial carcinoma (n=12)	6	6	0	
High-grade papillary urothelial carcinoma (n=14)	6	3	5	
Total	13	19	18	

Table 3 shows the distribution of p53 staining scores across different histological grades of UC. The table shows that, out of 24 invasive UCs, the maximum cases (15/24) showed a 4+ staining score, while in low-grade papillary UC and high-grade papillary UC, no case showed a p53 score of 4+. A significant correlation was seen with a p-value of 0.00925 on statistical analysis. 

**Table 3:** Association between histological grade and p53 staining percentage of positive cells ^1^Chi-squared test *: significant (p<0.05)

Histological grade	p53 staining score					P-value^1^
	0 (n=12)	1+ (n=0)	2+ (n=13)	3+ (n=10)	4+ (n=15)	
Invasive urothelial carcinoma (n=24)	0	0	4	5	15	0.009225*
Low-grade papillary urothelial carcinoma (n=12)	6	0	6	0	0	
High-grade papillary urothelial carcinoma (n=14)	6	0	3	5	0	
Total	12	0	13	10	15	

E-cadherin expression

Table 4 gives information on the distribution of cases based on H-score grading by E-cadherin staining. The H-score is a method used to evaluate the intensity of IHC staining by E-cadherin and was calculated using the formula H-score=((0×% no visible E-cadherin expression)+(1×% faint expression)+(2×% full-thickness non-circumferential expression)+(3×% full-thickness circumferential expression), with the overall score ranging from 0 (negative) to 300 (100% strong staining)). A low H-score was defined as an H-score value of <100, while a high H-score was defined as an H-score of >100. There were 28 (56%) cases that had high H-score, while the remaining 22 (44%) cases showed low H-score. 

**Table 4:** Case distribution according to grading by E-cadherin staining H-score ^1^Low H-score: H-score <100 ^2^High H-score: H-score >100

Grading (according to H-score)	No. of cases	Percentage
Low^1^	22	44
High^2^	28	56
Total	50	100

Table 5 shows how E-cadherin staining varies across different histological grades of UC. In histologically 24 cases of invasive UC, 22 cases showed low E-cadherin scores, while only two cases showed high E-cadherin scores. In histologically 12 cases of low-grade papillary UC and 14 high-grade papillary UC cases, all were shown high E-cadherin scores. On statistical analysis, a statistically significant difference was noted with a p-value of <0.001. It is evident from the table that invasive UC tends to have predominantly low E-cadherin staining H-scores, while low-grade papillary UC and high-grade papillary UC both predominantly exhibit high E-cadherin staining H-scores.

**Table 5:** Association between histological grade and E-cadherin percentage of positive cells ^1^Chi-squared test *: significant (p<0.05)

Histological grade	E-cadherin staining H-score		P-value^1^
	Low (n=22)	High (n=28)	
Invasive urothelial carcinoma (n=24)	22	2	<0.001*
Low-grade papillary urothelial carcinoma (n=12)	0	12	
High-grade papillary urothelial carcinoma (n=14)	0	14	
Total	22	28	

## Discussion

Bladder cancer is the most common malignancy affecting the urothelial tract, the lining of the urinary system. As the ninth most frequently diagnosed cancer worldwide, with over 400,000 new cases diagnosed annually [12], it presents a significant health challenge and highlights the need for continued research and improved approaches to prevention and treatment. UC comprises the majority of urinary tract malignancies that are routinely diagnosed on H&E‑stained sections. IHC plays a diagnostic role in UCs in distinct clinical settings, such as metastatic involvement of the bladder or in the diagnosis of metastatic lesions with unknown primary. UC is a recurrent neoplasm that progresses to an infiltrating, very aggressive disease in a significant number of cases. The study was undertaken at the Department of Pathology, Maharani Laxmi Bai Medical College, Jhansi, India. In this study, we studied the immunoexpression of p53 and E-cadherin in low-grade, high-grade, and invasive UC.

In our study, the largest group of cases, 14 (28%), fell within the 61-70 age range, followed by 11 cases (22%) in the 51-60 age range. The mean age of our cohort was 58.18±12.99 years, indicating that the majority of cases were over 55 years old. Our findings align with those of Nassa and Mahadevappa [13], who also found the largest group (17 cases) to be within the 61-70 age range, with only one case occurring below 30 years of age and a mean age of 63.38 years. Similarly, Nakanishi et al. [14] reported a patient age range of 34-84 years, with a median age of 66 years. Stephenson et al. [15] also observed a mean age of 70 years at initial diagnosis for all cases, further supporting the trend of bladder cancer predominantly affecting older individuals. In this study, the majority of the cases (76%) were males. The male-to-female ratio was 3.17:1. Results correlate with the study by Nassa and Mahadevappa [13] in which the male-to-female ratio was 6.14:1 and Nakanishi et al. [14] in which 73.4% of patients were males. 

Maximum cases in our study were from rural areas as our tertiary care center is surrounded by rural areas. In most of the cases, 24 (48%) were invasive UC followed by 14 (28%) cases of high-grade papillary UC. Only 12 (24%) cases were low-grade UC. In most of the cases, 26 (52%), muscle involvement was absent. 

Our study results are consistent with those of Nassa and Mahadevappa [13], who also observed a high prevalence of p53 overexpression in bladder cancer. In their analysis of 50 cases, 49 (98%) exhibited positive p53 overexpression, defined as staining in more than 10% of cells, while only one (2%) case was negative. Furthermore, their study demonstrated that the majority of cases displayed strong intensity of p53 overexpression, with 46% showing 3+ intensity and 48% exhibiting staining in over 75% of cells, corresponding to a score of 4+.

Thirteen out of 24 cases with histologically invasive UC showed strong p53 positivity and 10 cases showed moderate p53 staining. Weak p53 staining intensity was seen in just one case. Six cases had weak p53 staining and six cases had significant staining in low-grade UC.

In the study by Nassa and Mahadevappa [13], 40.7% of non-invasive tumors (11 cases) displayed a 3+ intensity of p53 overexpression, while 44.4% (12 cases) showed a score of 4+. Notably, 52.2% of invasive tumors (pT1 and above) also exhibited both a 3+ intensity and a 4+ score. However, the authors did not find a statistically significant correlation between pathological stage and p53 overexpression.

A study by Khurshid et al. [16] investigated p53 protein expression in UC, revealing a significant difference between high-grade and low-grade tumors. It found a very high prevalence (95.3%) of p53 overexpression in high-grade UC, detected through IHC analysis. Among these p53-positive tumors, the majority (74.4%) exhibited strong expression, with over half of the cells showing nuclear staining for p53. A smaller proportion (23.3%) showed moderate expression, with 10-50% of cells positive. Only one case (2.3%) demonstrated weak positivity, and none were entirely negative for p53 expression. In contrast to high-grade tumors, p53 overexpression was observed in a lower percentage (54.5%) of low-grade UC cases. The level of p53 expression also varied within this group. A minority (13.6%) showed strong expression, while a moderate proportion (31.8%) exhibited moderate expression. Another group (31.8%) displayed weak expression, and a significant number of cases (18.2%) were entirely negative for p53 expression. 

This study suggests a potential correlation between p53 overexpression and the grade of UC, hinting at a possible role for p53 in UC development and progression. In this study of histologically 24 cases of invasive UC, 22 cases showed low E-cadherin scores, while only two cases showed high E-cadherin scores. Histologically 12 low-grade papillary UC cases and 14 high-grade papillary UC cases showed high E-cadherin scores. Statistically, a significant correlation was noted with a p-value of <0.001. 

The study by Lotfy et al. [17] revealed a significant inverse relationship between E-cadherin expression and tumor stage in bladder cancer (p=0.031). This means that as the tumor stage progressed, the expression of E-cadherin decreased. Furthermore, the study found a highly significant association between decreased E-cadherin expression and high-grade tumors compared to low-grade tumors (p=0.002), suggesting a potential role for E-cadherin as a marker of tumor aggressiveness and progression.

Our findings regarding E-cadherin expression align with those of Esmail et al. [18], who reported a significantly higher positive expression in non-muscle-invasive bladder cancer (NMIBC) (65%) compared to muscle-invasive cases (10%). This difference was statistically significant (p<0.001). Similarly, our results echo those of Xie et al. [19], who demonstrated a significant correlation between decreased E-cadherin expression and both advanced pathological T stage and higher tumor grade in bladder cancer. This suggests a potential role for E-cadherin as a marker of tumor aggressiveness and progression. Ismail et al. [20] further support this notion, indicating that bladder tumor invasion is frequently associated with the downregulation of E-cadherin expression. They also identified a significant correlation between reduced E-cadherin levels and higher tumor grade. Additionally, Otto et al. [21] have highlighted the prognostic value of E-cadherin in bladder cancer, noting its absence in progressive cases. This emphasizes the importance of E-cadherin as a potential biomarker for predicting disease progression. In line with these findings, Paliwal et al. [22] also observed a statistically significant correlation between reduced E-cadherin expression and both tumor stage and grade (p<0.001 and p<0.01, respectively).

Taken together, these studies, along with our own, strongly suggest that E-cadherin plays a critical role in bladder cancer progression. Its reduced expression is a consistent marker of higher tumor stage, grade, and invasiveness.

Limitations

The limitations inherent in the present study may impede the generalizability of the findings and necessitate further investigation to confirm the validity of these results in a broader context. These limitations include the following: Firstly, a relatively small sample size (n=50) may not adequately represent the broader population of bladder cancer patients, potentially limiting the statistical power of the analysis and the ability to generalize the findings to a wider range of patients with diverse clinical characteristics. Secondly, another limitation of this study would be its single-center design. This might limit the generalizability of its findings to diverse populations and healthcare settings because there could be regional differences in demographic characteristics of patients, clinical practices, and available resources, among other factors, that may have altered the outcomes of this study.

To overcome these limitations and strengthen the results, a prospective study with the following characteristics is recommended: The first is a larger and more representative patient cohort to enhance the statistical power of the analysis and ensure that the findings apply to a wider range of bladder cancer patients. Next, multi-center studies conducted across various institutions, patients having different population characteristics, and healthcare practices can increase the generalizability of the findings. Lastly, meta-analyses combining data from multiple studies, including single-center studies, can bring out a more comprehensive understanding of p53 and E-cadherin expression in UC. 

Such a study design would provide more robust insights into the role of p53 and E-cadherin expression in bladder cancer progression and potentially inform the development of improved diagnostic and prognostic markers that could guide personalized treatment decisions and ultimately improve patient outcomes.

## Conclusions

Given the association between high p53 protein expression and aggressive tumor behavior, a more aggressive therapeutic approach is warranted for patients diagnosed with non-muscle-invasive UC (high-grade papillary UC) of the bladder who exhibit high scores and intensities of p53 protein upon IHC evaluation. This proactive treatment strategy is particularly relevant in developing countries, where the prevalence of tobacco smoking and, consequently, UC is notably increasing. IHC analysis of p53 offers a cost-effective and widely accessible method for identifying these high-risk patients, facilitating timely intervention, and potentially improving overall survival rates.

Conversely, the downregulation or loss of E-cadherin expression has been identified as a significant predictor of more advanced clinical stages and an elevated risk of metastasis in bladder cancer. This decreased expression is also frequently observed in tumors that have invaded the muscle layer and exhibit higher histological grades. By recognizing the correlation between low E-cadherin levels and aggressive tumor behavior, clinicians can further refine their treatment approach and potentially recommend more intensive therapies for patients with such characteristics.

The integration of p53 and E-cadherin expression data into clinical decision-making processes holds the potential to enhance risk stratification, allowing for a more personalized and targeted approach to bladder cancer treatment. By identifying patients with high-risk profiles early in the course of the disease, clinicians can implement more aggressive treatment strategies upfront, potentially improving the chances of achieving long-term disease control and survival for these individuals.
